# Objective parameters aid the prediction of fistulas in pancreatic surgery

**DOI:** 10.3892/etm.2014.1829

**Published:** 2014-07-07

**Authors:** KLAUS FELIX, ANNA SCHUCK, MATTHIAS M. GAIDA, ULF HINZ, DMITRIY DOVZHANSKIY, JENS WERNER

**Affiliations:** 1 Department of General Surgery, University of Heidelberg, Heidelberg D-69120, Germany; 2 Institute of Pathology, University of Heidelberg, Heidelberg D-69120, Germany

**Keywords:** pancreatic fistula, predictive markers, postoperative complications, chronic pancreatitis, carcinoma of the pancreas

## Abstract

Insufficiency of pancreatic anastomosis with leakage from the pancreatic stump and the development of fistulas account for the majority of surgical complications following pancreatic resection, which are often life threatening. The cause of pancreatic fistulas of the remnant tissue on a molecular level remains unclear. Thus, the aim of the present study was to investigate risk factors associated with postoperative pancreatic fistula (POPF) formation and to define parameters that may predict the resection outcome. Pancreatic resection margins were selected from 31 patients, including 16 individuals without and 15 patients with POPF, to analyze the degree of fibrosis, lipomatous atrophy, inflammatory activity and infiltration. Wound healing factors were assessed by luminex technology using tissue homogenates, while the distribution *in situ* was assessed using immunohistochemistry. Increased chronic inflammatory infiltration, a higher degree of fibrosis and a reduction in lipomatous atrophy were observed in the samples without anastomotic fistulas. Multiplex analysis of 38 wound healing factors demonstrated significantly higher levels of interleukin (IL)-6, -8 and -12, glucagon-like peptide-1 and matrix metalloproteinase (MMP)-1, -2, -3 and -12 in the group without fistulas, while lower concentrations of IL-10, IL-17 and gastric inhibitory polypeptide were observed. Therefore, the observations of the present study indicated that increased inflammatory infiltration and inflammatory activity, as well as higher concentrations of proinflammatory cytokines and higher MMP levels at the resection margins, predisposed individuals to a lower fistula incidence rate following pancreatic resection.

## Introduction

Pancreatic fistula and anastomotic leakage are major complications following pancreatic head resection or distal pancreatectomy (1,2), which are often associated with subsequent dangerous infectious complications, including peritonitis and sepsis (3). Therefore, these complications represent the leading cause of mortality following pancreatic resection. A primary aim during pancreatic surgery is to reduce the incidence rate of fistulas and thus the mortality rate. Although the mortality rate has been reduced in centers of excellence to <5% (4), the morbidity rate remains high (2). The outcome of pancreatic surgery, including the leakage rate, is dependent on the experience of the center and the individual surgeon, as well as the surgical technique. However, there are also patient-specific and tissue-determined risk factors for pancreatic leakage or fistula, which include the soft tissue texture of the pancreas (3) and a small pancreatic duct of <3 mm (5–7). While these risk factors are widely accepted, to date, no histological or molecular factors have been identified. Postoperative pancreatic fistulas (POPFs) are a consequence of inadequate regeneration of the pancreatic tissue following resection and insufficient wound healing of the pancreatic remnant. Wound healing is a complex process involving a coordinated interplay of cells, extracellular matrix and numerous regulatory mediators. It also includes an organized stimulation of angiogenesis, fibroblast proliferation with stimulation of extracellular matrix and the growth of epithelial tissue. The process can be divided into three well-defined phases: Inflammatory, proliferative and tissue remodeling phases (8). In each phase there are regulated and regulatory mediators. Impaired wound healing has been hypothesized to be caused by the dysfunctional coordination of wound healing mediators, which may result in the overexpression or suppression of certain factors (9). Impaired healing of chronic wounds is known to be mediated by the dysregulation of numerous factors (10,11), including pro- and anti-inflammatory cytokines, angiogenesis-associated proteins, proteins associated with diabetic conditions and matrix metalloproteinases (MMPs) that remodel damaged tissue.

The aim of the present study was to investigate whether the tissues of patients with POPF may be predicted and differentiated from those without complications using the molecular composition of the pancreatic resection margins.

## Materials and methods

### Patients

Between August 2008 and November 2009, 435 pancreatic head resections and distal pancreatectomies were performed at the Department of General Surgery of the University of Heidelberg (Heidelberg, Germany). In total, 12% of these resections developed a pancreatic fistula. In this study, 31 patients were selected randomly. Indications for their pancreatic resection were malignant pathologies (n=25) or chronic pancreatitis (n=6). The tissue with the resection margins was preserved in each case. Informed consent was obtained preoperatively from all the patients (males, 19; females, 12; mean age, 63.8 years; age range, 48–79 years) and approval was obtained from the designated Ethics Commission of the University of Heidelberg. Pancreaticoenteric anastomosis was performed using the same method for all the patients, as previously described (12). Experienced surgeons performed all the resections. Patients without a fistula (n=16), as well as those with POPF (n=15), were selected according to the criteria of the International Study Group on Pancreatic Fistula definition (1,2). POPF is the failure of healing/sealing a pancreatic-enteric anastomosis or a parenchymal leak not directly associated with an anastomosis, and was defined as a drain output of any measurable volume on or following day three postoperatively, with an amylase content greater than three times the serum amylase activity. According to the clinical impact on the patient’s hospital course, three different grades of POPF may be differentiated (grades A, B and C) (4,13).

### Tissue

Tissue samples were immediately shock frozen in liquid nitrogen and fixed in formalin for paraffin embedding. For multiplex protein analysis and immunohistochemistry (IHC), all the cryopreserved samples were sectioned into aliquots and stored at −80°C until required for further analysis.

### Histological analysis

Paraffin-embedded pancreatic tissue sections were cut into 3–4 μm slices using a microtome (Leica, Wetzlar, Germany), deparaffinized, rehydrated in graded alcohols, stained with hematoxylin and eosin, then dehydrated and examined using an Axioplan 2 Imaging, Zeiss light microscope (Carl Zeiss, Goettingen, Germany). An experienced pathologist investigated the samples for their histological grade of fibrosis, lipomatous atrophy, inflammatory activity, inflammatory infiltration and necrosis using a semi-quantitative scoring system developed in consultation with a specialized pathologist (Table I).

### Multiplex protein analysis

Tissue sample concentrations of the selected wound healing mediators were determined using multiplex protein arrays (Biorad, Biorad Laboratories GmbH, Munich, Germany), enabling quantification of all the parameters in one sample. The factors were assessed in four group panels: (i) The cytokine panel, using the Bio-Plex Pro Human Cytokine 17-plex panel (Bio-Rad Laboratories, Inc., Munich, Germany) composed of interleukin (IL)-1β, -2, -4, -5, -6, -8, -10, -12, -13 and -17, granulocyte colony-stimulating factor (G-CSF), granulocyte-macrophage colony-stimulating factor, interferon-γ, monocyte chemotactic protein-1, macrophage inflammatory protein-1β and tumor necrosis factor (TNF)-α; (ii) the angiogenesis panel, using the Bio-Plex Pro Human Angiogenesis 9-plex panel (Bio-Rad Laboratories, Inc.) composed of vascular endothelial growth factor (VEGF), angiopoetin-2, follistatin, G-CSF, hepatocyte growth factor, IL-8, platelet-derived growth factor-BB, platelet endothelial cell adhesion molecule-1 and leptin; (iii) the diabetes panel, using the Bio-Plex Pro Human Diabetes 9-customized plex (Bio-Rad Laboratories, Inc.) consisting of c-peptide, gastric inhibitory polypeptide (GIP), ghrelin, glucagon-like peptide-1 (GLP-1), insulin, leptin, plasminogen activator inhibitor-1, TNF-α and IL-6; and (iv) the MMP panel (MMP-kit; R&D Systems, Abingdon, UK), including MMP-1, -2, -3, -7, -8, -9, -12 and -13.

In order to quantify the 38 parameters, 100 mg frozen tissue were cut with a cryotome in thin serial section slices (Leica CM3050 S Cryostat; Leica Biosystems, Nussloch, Germany) and subsequently ground with a mortar in liquid nitrogen. The powder was transferred into prechilled 15-ml Falcon tubes and 500 μl lysis buffer (Bio-Rad Laboratories, Inc.) was added. The suspension was subjected to a 30 sec sonification step on ice (amplification 80%; 0.99 kJ; Sonoplus; Bandelin, Berlin, Germany) and subsequently centrifuged at 16,000 × g for 10 min. Supernatants were collected and the total protein concentration was determined using the bicinchoninic acid assay (Pierce Biotechnology, Inc., Rockford, IL, USA). For multiplexing, the samples were adjusted with a Sample Diluent Buffer (Bio-Rad Laboratories, Inc.) to a final protein concentration of 2 mg/ml.

Multiplexing was performed in accordance with the manufacturer’s instructions. Briefly, beads coated with anti-human antibodies against the examined biomarker antigen were mixed with 200 μl each diluted patient sample (50 μl supernatant and 150 μl dilution buffer) and then incubated for 30 min. Following a wash cycle, a biotinylated detection antibody specific to another epitope of the examined biomarker-antigen was added and the samples were incubated for an additional 30 min. A second wash cycle was then performed, after which streptavidin-phycoerythrin was added to the beads and a third wash cycle was conducted, followed by incubation for 10 min. Following the removal of excess conjugate, the bead mixture was analyzed using a BioPlex 200 System (Bio-Rad Laboratories, Inc.).

Raw data were initially measured as the relative fluorescence intensity and then converted to a fluorescence ratio using predyed internal standard beads (Bio-Rad Laboratories, Inc.). A series of calibrators were analyzed with the patient samples to convert the fluorescence ratio to international units per milliliter. All the samples were measured in triplicate. Overlapping analytes from different panels were analyzed and a combined evaluation was performed. Standard curves and concentrations were calculated using Bioplex Manager 6.1 software (Bio-Rad Laboratories, Inc.).

### IHC

For IHC detection, polyclonal antibodies against IL-6 (1:500), IL-8 (1:25) and VEGF (1:100; Abcam, Cambridge, UK) and monoclonal antibodies against MMP-1 (1:100; Merck Calbiochem, Darmstadt, Germany) and MMP-2 (1:100; Dianova, Hamburg, Germany) were used. Frozen sections were cut into 10-μm thick slices using a Leica cryotome at −20°C. The IHC staining protocol was performed as previously described (14). Tissue sections were scored semi-quantitatively and the IHC scores were assigned a numerical value in consultation with the pathologist. The staining distribution and intensity were graded according to the following criteria. Distribution of the staining: 0, no staining; 1, focal staining; 2, moderate staining; and 3, diffuse staining. Intensity of the staining: 0, none; 1, weak intensity; 2, moderate intensity; and 3, strong intensity. All the slides were examined by an experienced pathologist.

### Statistical analysis

Continuous variables are expressed as the median and range. The non-parametric Mann-Whitney U test was used to compare the continuous variables between the two study groups. Categorical variables are presented as absolute and relative frequencies, and comparisons of the categorical variables between the two study groups were performed using Fisher’s exact test. The exact Cochran-Armitage trend test was used to compare three or four categories. P<0.05 was considered to indicate a statistically significant difference, and all the tests used were two-sided.

Statistical analysis was performed using the GraphPad Prism 5 software (GraphPad Software, Inc., San Diego, CA, USA) and SAS software (Release 9.1; SAS Institute, Inc., Cary, NC, USA).

## Results

### Histology

**Figure 1:**
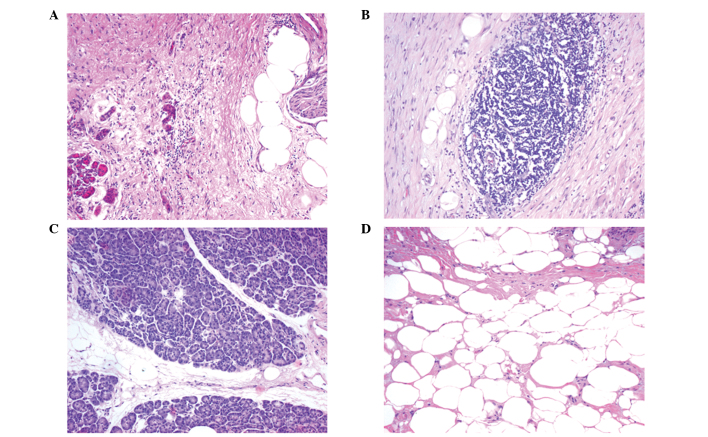
Original grading examples of the histological analysis of the resection edges (hematoxylin and eosin stain; magnification, ×100). (A) Lipomatous atrophy grade 1; (B) lipomatous atrophy grade 3; (C) chronic inflammatory infiltration grade 1; and (D) chronic inflammatory infiltration grade 3.

A histological comparison of the tissue sections derived from the groups with and without a fistula revealed differences in the grade of fibrosis, lipomatous atrophy, inflammatory activity, inflammatory invasion and necrosis. Representative histological sections for lipomatous atrophy, chronic inflammatory infiltration and their grading are presented in Fig. 1.

### Fibrosis

**Figure 2:**
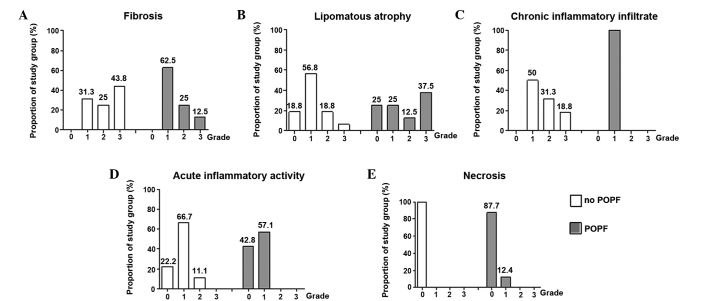
Histopathological scores were assessed with numerical values (0–3) and expressed as the percentage proportion of the experimental group for (A) fibrosis, (B) lipomatous atrophy, (C) inflammatory infiltration, (D) inflammatory activity and (E) necrosis.

Fibrosis was observed in all the samples to a certain extent. The majority (62.5%) of the patients with a fistula had weak periductal fibrosis (grade 1) compared with 31.3% in the group without a fistula (Fig. 2A). However, extensive fibrosis was more frequently observed in the samples from the group without a fistula (43.8%) compared with the group with a fistula (12.5%).

### Lipomatous atrophy

Lipomatous atrophy was observed in the two groups with and without fistulas; however, the distribution and extent of lipomatous atrophy differed between the groups. In the group without a fistula, the majority of the samples were classified as little (56.3%) and rarely with severe lipomatous atrophy (6.3%), whereas in the fistula group, the majority of the samples had severe (37.5%) or little lipomatous atrophy (25%). These results revealed more extensive lipomatous atrophy in the resection margins from patients developing a pancreatic fistula (Fig. 2B).

### Chronic inflammatory infiltration

In order to determine the grade of chronic infiltration, the overall infiltration of inflammatory cells (leukocytes, macrophages and plasma cells) was investigated. All the resection edge tissues of the fistula group exhibited little (grade 1) chronic inflammatory infiltration when compared with the tissues without a fistula, which exhibited little (50% grade 1), moderate (31.3% grade 2) or even severe chronic inflammatory infiltration (18.8% grade 3). These observations indicated an overall increased chronic inflammatory infiltration rate in the samples without a fistula (Fig. 2C).

### Acute inflammatory activity

To classify the degree of inflammatory activity, the quantity of neutrophil granulocytes was evaluated. In the fistula group, no neutrophil granulocytes were observed in 42.8% of the individuals, while 57.1% of the group exhibited little acute inflammatory activity. Furthermore, none of the patients with a fistula exhibited moderate or severe inflammatory activity (grade 2 or 3). However, patients without a fistula exhibited no (22.2%), little (66.7% grade 1) or even severe acute inflammatory infiltration (11.1% grade 2). Similar to chronic inflammatory infiltration, slightly increased acute inflammatory activity was observed in the group without fistulas (Fig. 2D).

### Necrosis

Of all the examined samples, only 12.4% showed single cells with necrosis (grade 1) in the fistula group, while no necrosis was observed in any case from the fistula free group (Fig. 2E).

### Proteins involved in the wound healing process

Quantitative multiplex analysis of the resection margins from all the patients with pancreatic head resections or distal pancreatectomies was performed for 38 proteins. The analyzed factors were selected due to their essential roles in the wound healing process and their regulatory functions in inflammation, neovascularization, glucose metabolism and tissue remodeling. The results of the individual parameters are summarized in Table II.

In the cytokine panel, significantly higher concentrations of the proinflammatory cytokines, IL-6, -8 and -12, were observed in patients without a fistula, while significantly higher concentrations of the anti-inflammatory cytokines, IL-10 and -17, were observed in the fistula group. Analysis of neovascular factors revealed elevated values for all the parameters in the fistula-free group; however, the values were not statistically significant.

In the diabetes panel, in addition to IL-6, the GLP-1 concentration was found to be higher in patients without a fistula, whereas the GIP level was observed at significantly higher concentrations in the fistula group.

MMP analysis revealed highly expressed profiles for MMP-1, -2, -3 and-12 in the group without a fistula (Table II).

### IHC

**Figure 3:**
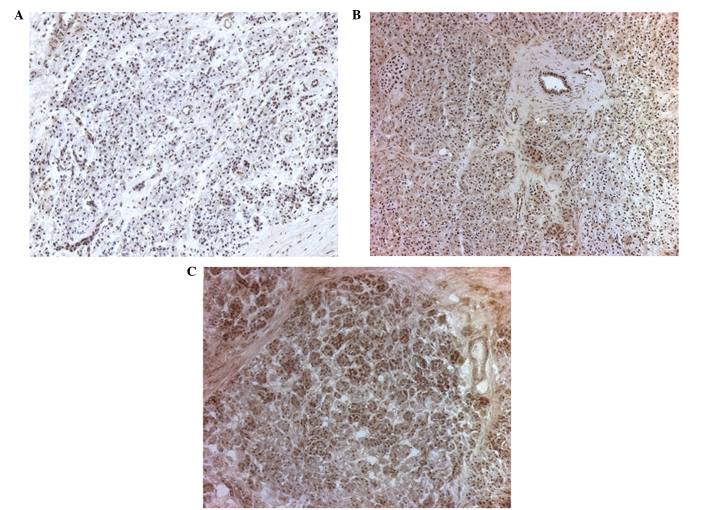
Original grading examples from the IHC analysis of the resection margins for MMP-1 (magnification, ×100). (A) Intensity grade 1 (weak); (B) intensity grade 2 (moderate); and (C) intensity grade 3 (strong). MMP, matrix metalloproteinase; IHC, immunohistochemistry.

Based on the multiplex analysis, five factors, including IL-6, IL-8, VEGF, MMP-1 and MMP-2, were additionally analyzed using IHC. A total of 10 resection margins from the two groups were stained for the five factors and semi-quantitative scoring was performed. The staining distribution and intensity were numerically graded and evaluated by an experienced pathologist. A representative example of the MMP-1 intensity grading is shown in Fig. 3. The scoring data were statistically analyzed using Fisher’s exact test and the Cochran-Armitage trend test. However, statistically significant differences were only obtained in the tests for IL-8 and MMP-1 (Table III).

## Discussion

Despite major progress in pancreatic surgery, morbidity rates following pancreatic resection remain high, with pancreatic fistulas being the most challenging postoperative complication (2). The present study provides a comprehensive analysis of the morphological and biochemical parameters associated with healing of the pancreatic remnant following resection.

Histological analysis of the tissues demonstrated a high degree of fibrosis, elevated inflammatory activity and higher inflammatory infiltration, as well as an absence of lipomatous atrophy in the pancreatic tissue, correlating with a low incidence of pancreatic fistulas and vice versa. These results are in accordance with previous studies that found a non-fibrotic fragile pancreas is likely to predispose individuals to the development of POPF (5–7). Fibrotic pancreatic tissue is easier to handle for surgeons, while soft pancreatic tissue is difficult to sew, which partially explains the increased leakage rates of soft pancreatic tissue. However, in addition to the technical factor, tissue-remnant factors appear to have an important role in the process of healing, as demonstrated in the present study.

Chronic inflammatory infiltration by macrophages, B-cells and T-cells was more frequently observed in patients without a fistula. The acute inflammatory activity (level of neutrophil granulocytes) was slightly elevated following successful resections. These observations correlate with the results from the biochemical analysis using quantitative multiplex protein analysis. The panel of 38 wound healing key proteins revealed higher concentrations of proinflammatory factors, including IL-6, -8 and -12 and MMP-1, -2, -3 and -12, and lower concentrations of IL-10 (anti-inflammatory), IL-17 (MMP modulating), and GIP in the resection margins of patients without any incidence of fistula formation compared with those tissues from patients with POPF.

A pancreatic fistula or anastomotic insufficiency following pancreatic resection should be considered as insufficient wound healing, which in a normal course, passes through four defined phases: Bleeding, inflammatory, proliferative and remodeling phases (8). These phases have to be in perfect equilibration and are regulated by cytokines and other mediators. Under- or overexpression, as well as continuous ongoing expression, of these mediators is known to induce failure of wound healing, which is observed in chronic inflammatory diseases, including chronic ulcers (9–11). In the present study, the increase in chronic inflammation and proinflammatory cytokines (IL-6, -8 and -12), as well as the decrease in anti-inflammatory cytokines (IL-10), was shown to correlate with a decreased rate of pancreatic fistula. Thus, mild to moderate, but not severe inflammation, appears to be a major factor involved in the healing of pancreatic remnant and anastomosis. The present study assessed the initial intraoperative situation of the tissue and in this regard mirrored the tissue condition prior to the healing process. IL-6 is a proinflammatory cytokine that exhibits elevated levels in the first hours following injury. The level of IL-6 correlates with acute phase reactions, and the cytokine promotes the transition from unspecific to specific immune defense (15–17). IL-8, also a potent proinflammatory chemokine, recruits neutrophil granulocytes and T-cells to the infection site (9,11,18). The effects of IL-6 and IL-8 explain their positive effects on the healing process. The main function of the anti-inflammatory cytokine, IL-10, is the termination or limitation of inflammatory responses by suppressing inflammatory reactions and cytokine production and inhibiting macrophage activity. Higher concentrations of the anti-inflammatory IL-10 in non-healing anastomosis may reinforce a weaker immune defense of the tissue (19,20).

Factors associated with angiogenesis or diabetes did not exhibit statistically significant differences, with the exception of two incretins, GLP-1, which was significantly higher in the complication free group (without POPF), and GIP, which had increased levels in the tissues with POPF. GIP, also known as glucose-dependent insulinotropic peptide GIP, similar to GLP-1, is expressed shortly following ingestion and exerts effects on β-islet cells. Whether the higher levels of GIP in the POPF group are a compensatory effect for lower GLP-1 levels is unclear. GLP-1 and GIP are regarded as antidiabetic cytokines. It is well-known that diabetic metabolic conditions inhibit wound healing (10); however, no association was observed with the observations of the present study.

The remodeling phase of wound healing is dependent on MMPs, which degrade the extracellular matrix to aid cells migration and are engaged in remodeling processes in tissues (21–23). The remodeling processes, as well as MMP expression, are highly coordinated and maintained in a balance. In the present study, tissue samples of patients without fistulas were found to have higher concentrations of MMP-1, -2, -3 and -12 when compared with the tissues from patients with a fistula. Therefore, high expression levels of MMP-1, -2, -3, and -12 appear to promote adequate healing. The differential expression of MMPs in the pancreas of patients with and without fistulas may be further investigated using IHC.

The present study investigated the morphological and biochemical predictive factors for anastomotic complications following pancreatic resection. The histological results revealed a higher degree of fibrosis and increased inflammatory activity, as well as a lower degree of lipomatous atrophy, in the pancreatic resection margins of patients without pancreatic fistulas or anastomotic insufficiencies. Furthermore, the results from the protein profiling indicated that a low predisposition to inflammatory reaction results in lower concentrations of proinflammatory cytokines, including IL-6, -8 and -12, as well as high concentrations of anti-inflammatory cytokines, such as IL-10, and decreased expression levels of MMP-1, -2, -3 and -12. These conditions are associated with a higher postoperative complication and fistula incidence rate. Accordingly, the MMP modulating cytokine, IL-17, was found in higher concentrations when the healing of the pancreatic remnant failed.

The development of a preoperative or intraoperative test may eventually aid or assure the surgeons intraoperative decision of performing an anastomosis in individual critical cases where the pancreas texture is intraoperatively macroscopically complex to evaluate.

Furthermore, a predictive statement to patients with an increased risk of developing an anastomosis insufficiency or fistula is possible, leading to close meshed patient’s monitoring postoperatively. However, in order to develop a clinically practical assay to predict pancreatic fistulas, further studies investigating the role of inflammatory cytokines, chemokines and MMPs are required. The present study, to the best of our knowledge, is one of the only studies investigating this topic.

## Figures and Tables

**Table I:** Histological grading scores.

Grading	Fibrosis	Lipomatous atrophy	Inflammatory infiltration	Inflammatory activity	Microscopic necrosis
0	No	No	No	No	No
1	Periductal	Little	Little	little	Single cells
2	Periductal, intra- and interlobular	Moderate	Moderate	Moderate	Grouped necrosis
3	Extensive	Severe	Severe	Severe	Broad

**Table II:** Quantitative multiplexing protein analysis.

	No POPF, pg/mg total protein (n=16)			POPF, pg/mg total protein (n=15)			
			
Analyte	Min	Median	Max	Min	Median	Max	P-value^a^
IL-1β	2.2	59.0	2427	13.2	71.3	1665	0.7529
IL-2	2.44	2.4	318.7	2.4	2.4	1845	0.8615
IL-4	4.7	9.6	16.7	4.6	9.2	16.7	0.8260
IL-5	2.2	3.1	8.9	2.2	3.2	3.62	0.5990
IL-6	1.7	301.3	11705	0.7	64.6	20730	0.0345
IL-7	2.3	366	4579	36.9	1788	3116	0.3106^b^
IL-8	3.1	218	23614	2.7	46	1114	0.0029^b^
IL-10	2.3	8.2	26.9	69.5	881	1901	<0.0001^b^
IL-12	3.1	331	1191	3.3	10.4	33,93	0.0045^b^
IL-13	50.0	90	4553	49.5	103.5	4283	0.9538
IL-17	1.6	128.9	264.5	94.0	167.8	335	0.0399^b^
G-CSF	1.3	301.3	2796	1.3	65	2414	0.6050
IFN-γ	0.0	370.9	12585	0.0	659.5	2714	0.3099
GM-CSF	560.4	898	2892	757.8	1057	1466	0.0570
MCP-1	0.1	2882	34386	986.1	2901	23659	0.6334
MIP-1β	0.0	2033	8178	0.0	648.4	22307	0.1548
TNF-α	1.2	1.9	131.6	1.2	3.3	141	0.7136
Angiopoietin-2	21.8	205.6	648.1	0	61.3	11684	0.1382
Follistatin	64.7	272.9	782.4	41.0	200.3	801.2	0.2949
HGF	128.6	3216	10962	132.4	3385	9905	0.8279
PDGF-BB	8.3	21.6	381.7	2.9	17.0	158.6	0.3365
PECAM-1	10751	22656	22656	1528	18530	22656	0.2770
VEGF	3.6	38.5	226.4	1.0	49.2	176.8	0.3845
C-peptide	8.5	2691	2691	1.1	31.3	2691	0.0709
Ghrelin	2.0	11.6	485.9	2.0	71.2	287.8	0.1638
GIP	0.5	2.4	6.5	0.8	4.3	6.1	0.0225^b^
GLP-1	0.5	1053	1645	0.5	502.8	1409	0.0402^b^
Insulin	0.3	2027	2027	2.6	2027	2027	0.4873
Leptin	7.9	92.1	406.6	5.4	75.9	248.5	0.1015
PAI-1	130	1134	5712	37.2	421.8	2201	0.1149
MMP-1	1.0	44.69	914.8	9.886	33.58	46.91	0.0452^b^
MMP-2	1473	15924	23015	47.07	3344	21497	0.0049^b^
MMP-3	20.24	997.5	5747	0	159.8	1144	0.0012^b^
MMP-7	92.55	124.6	1034	95.11	113.1	136.2	0.0880
MMP-8	519.2	15319	88596	0	10490	169078	0.6494
MMP-9	3904	41120	5.26×10^6^	774	29880	5.45×10^6^	0.9842
MMP-12	189.0	197.8	238.7	3.249	191.9	218.2	0.0369^b^
MMP-13	82.84	105.9	240	73.6	103.6	333.5	0.9202

Concentrations of 38 wound healing analytes in the resection edge tissue following pancreatic surgery. The group without fistulas were compared with the fistula group using range, median and P-values.

a Mann-Whitney U-test. The two groups included patients with pancreas head resections and distal pancreatectomy.

b Statistically significant differences.

POPF, postoperative pancreatic fistula formation; IL, interleukin; G-CSF, granulocyte colony-stimulating factor; IFN, interferon; GM-CSF, granulocyte-macrophage colony-stimulating factor; MCP, monocyte chemotactic protein; MIP, macrophage inflammatory protein; TNF, tumor necrosis factor; HGF, hepatocyte growth factor; PDGF, platelet-derived growth factor; PECAM, platelet endothelial cell adhesion molecule; VEGF, vascular endothelial growth factor; GIP, gastric inhibitory polypeptide; GLP, glucagon-like peptide; PAI, plasminogen activator inhibitor; MMP, matrix metalloproteinase; Min, minimum; Max, maximum.

**Table III:** IHC analysis of the frequency and validation of the biochemical parameters.

	IL-6		IL-8		VEGF		MMP-1		MMP-2	
					
Parameter	Inten	Distrib	Inten	Distrib	Inten	Distrib	Inten	Distrib	Inten	Distrib
POPF										
**0**	0	0	0	0	0	0	1	1	0	0
**1**	3	3	0	1	3	3	4	4	5	1
**2**	5	7	6	9	5	7	5	5	4	9
**3**	2	0	4	0	2	0	0	0	1	0
No POPF										
**1**	2	5	0	6	3	6	2	3	1	1
**2**	4	5	9	4	7	4	4	7	8	9
**3**	4	0	1	0	0	0	4	0	1	0
P-value^a^	0.727	0.649	0.303	**0.057**	0.542	0.369	0.124	0.649	0.140	1.000
P-value^b^	0.558	0.649	0.303	**0.057**	0.719	0.369	**0.054**	0.469	0.276	1.000
Int. × distrb^a^	0.337		**0.015**		0.470		**0.017**		0.47	

Staining distributions and intensities were graded as described in the material and methods.

a Fisher’s exact test;

b exact Cochran-Armitage trend test in cases of three or four categories.

IL, interleukin; VEGF, vascular endothelial growth factor; MMP, matrix metalloproteinase; POPF, postoperative pancreatic fistula formation; Inten, intensity; Distrib, distribution; IHC, immunohistochemistry. The values in bold indicate statistically significant differences or close to being significant. Int × distrb is an additional scoring option in our analysis supporting the significance of the individual validations for intensity and for distribution.
